# Comment on “Vasoplegic syndrome following cardiothoracic surgery-review of pathophysiology and update of treatment options”

**DOI:** 10.1186/s13054-020-02854-1

**Published:** 2020-04-07

**Authors:** Jamel Ortoleva, Frederick C. Cobey

**Affiliations:** Tufts Medical Center Department of Anesthesiology and Perioperative Medicine, Boston, USA

We read with great interest the article by Bousse et al. on vasoplegic syndrome after cardiothoracic surgery [1]. It provides an excellent review of alternative pharmacologic interventions when vasodilatory shock becomes refractory to usual measures of catecholamines and vasopressin. In the article by Busse et al., the following statement is made: “In a protocol by Ortoleva et al., non-catecholamine therapy is recommended to begin at norepinephrine doses of 0.5μg/kg/min, which has been associated, at least in the distributive shock population, with an unacceptable level of mortality.” [1, 2]. We wish to provide a clarification on this reference to our algorithm on the management to vasoplegia [3].

Our article recommends the initiation of alternate therapy at the equivalent of 0.5 μg/kg/min of norepinephrine (or other agreed upon limit) and not when the norepinephrine dose by itself has reached that level. Our article also states that “The use of vasopressin, norepinephrine, or phenylephrine are left to the discretion of the clinician...” [3]. While we believe vasopressin is a valuable addition in vasodilatory shock, the recent price increase on vasopressin has sanctioned use at our and other institutions. Hence, we did not specifically mention the use of vasopressin and instead elected to use a norepinephrine equivalent dose of 0.5 μg/kg/min which, for example, could be 0.3 μg/kg/min of norepinephrine and 0.08 U/min of vasopressin, among other possible combinations.

The article by Sviri et al. is an observational study of medical intensive care unit (MICU) patients receiving either no vasopressors, less than 40 μg/min norepinephrine or at least 40 μg/min of norepinephrine [2]. A mortality of 84.3% in the MICU and 90% for the hospitalization was noted in patients receiving at least 40 μg/min of norepinephrine. However, this study notes that 43% of patients receiving high dose norepinephrine were also receiving vasopressin versus 14% in the low dose norepinephrine group. Furthermore, no patient weights were available. Hence, this article cannot be used to justify a certain weight-based threshold of high or low dose catecholamine vasopressor support because no mention is made of patient weight. Multiple articles on the use of high dose weight based norepinephrine in intensive care units exist (0.9 μg/kg/min or more and 1 μg/kg/min or more), and 0.5 μg/kg/min of norepinephrine is not used as a cut off to define “high dose vasopressors” [4, 5].

In conclusion, we wish to clarify that our article uses norepinephrine equivalent doses which can include vasopressin to allow clinicians the freedom to select therapy that is in accordance with individual institutional guidelines.

## Authors’ response

### Response to “Comment on Vasoplegic Syndrome Following Cardiothoracic Surgery-Review of Pathophysiology and Update of Treatment Options”

Laurence W. Busse, M.D., FCCM

We thank Dr. Ortoleva and Dr. Cobey for their clarifying letter and acknowledge that the definition of “high dose vasopressors” is variable and not quantitative. It should also be pointed out that individual patients may display the side effects of catecholamine therapy at different thresholds further aggravating the possibility of a consensus definition. The study by Sviri et al. is just one of many analyses which attempt to associate outcomes with doses of vasopressor therapy. Many authors describe a catecholamine threshold of 0.5 mcg/kg/min which was acknowledged by Ortoleva et al. as the point at which alternate therapy should begin [6–8]. Alternatively, “high-dose” therapy has been defined as the need for rescue therapy with vasopressin [9, 10]. Moreover, some thresholds are weight-based [5, 11, 12] while others are not [13, 14]. There can be no doubt, however, that high doses of catecholamines are associated with adverse events, including mortality [15] and organ failure [16]. Notwithstanding some of the economic pressures that Ortoleva et al. are right to point out (including the cost of vasopressin), it is our opinion that de-catecholaminization at lower cumulative doses may mitigate some of these adverse effects seen from high-dose therapy [17]. The non-catecholamine agents highlighted in our manuscript, including vasopressin, are distinctly different from catecholamines, and should be separately and thoughtfully deployed in the right circumstances.

## Data Availability

This article is not a study and hence there is no data or material.

## References

[1] Busse LW, Barker N, Petersen C (2020). Vasoplegic syndrome following cardiothoracic surgery-review of pathophysiology and update of treatment options. Crit Care.

[2] Sviri S, Hashoul J, Stav I, van Heerden PV (2014). Does high-dose vasopressor therapy in medical intensive care patients indicate what we already suspect?. J Crit Care.

[3] Ortoleva JP, Cobey FC (2019). A systematic approach to the treatment of vasoplegia based on recent advances in pharmacotherapy. J Cardiothorac Vasc Anesth.

[4] Auchet T, Regnier M-A, Girerd N, Levy B. Outcome of patients with septic shock and high-dose vasopressor therapy. Ann Intensive Care. 2017;7(1) Available from: http://annalsofintensivecare.springeropen.com/articles/10.1186/s13613-017-0261-x. [cited 2018 Aug 17]. 10.1186/s13613-017-0261-x PMC5397393 28425079

[5] Brown SM, Lanspa MJ, Jones JP, Kuttler KG, Li Y, Carlson R (2013). Survival after shock requiring high-dose vasopressor therapy. Chest..

[6] Ortoleva JP, Cobey FC: A systematic approach to the treatment of vasoplegia based on recent advances in pharmacotherapy. (1532–8422 (Electronic)). 2019. 10.1053/j.jvca.2018.11.025 30598380

[7] Benbenishty J, Weissman C, Sprung CL, Brodsky-Israeli M, Weiss Y (2011). Characteristics of patients receiving vasopressors. Heart Lung.

[8] Dunser MW, Mayr AJ, Ulmer H, Knotzer H, Sumann G, Pajk W, Friesenecker B, Hasibeder WR (2003). Arginine vasopressin in advanced vasodilatory shock: a prospective, randomized, controlled study. Circulation.

[9] Dunser MW, Mayr AJ, Ulmer H, Ritsch N, Knotzer H, Pajk W, Luckner G, Mutz NJ, Hasibeder WR (2001). The effects of vasopressin on systemic hemodynamics in catecholamine-resistant septic and postcardiotomy shock: a retrospective analysis. Anesth Analg.

[10] Luckner G, Dunser MW, Jochberger S, Mayr VD, Wenzel V, Ulmer H, Schmid S, Knotzer H, Pajk W, Hasibeder W (2005). Arginine vasopressin in 316 patients with advanced vasodilatory shock. Crit Care Med.

[11] Park BK, Shim TS, Lim CM, Lee SD, Kim WS, Kim DS, Kim WD, Koh Y (2005). The effects of methylene blue on hemodynamic parameters and cytokine levels in refractory septic shock. Korean J Intern Med.

[12] Castro R, Regueira T, Aguirre ML, Llanos OP, Bruhn A, Bugedo G, Dougnac A, Castillo L, Andresen M, Hernandez G (2008). An evidence-based resuscitation algorithm applied from the emergency room to the ICU improves survival of severe septic shock. Minerva Anestesiol.

[13] Jenkins CR, Gomersall CD, Leung P, Joynt GM (2009). Outcome of patients receiving high dose vasopressor therapy: a retrospective cohort study. Anaesth Intensive Care.

[14] Russell JA, Walley KR, Singer J, Gordon AC, Hebert PC, Cooper DJ, Holmes CL, Mehta S, Granton JT, Storms MM (2008). Vasopressin versus norepinephrine infusion in patients with septic shock. N Engl J Med.

[15] Bassi E, Park M, Azevedo LC (2013). Therapeutic strategies for high-dose vasopressor-dependent shock. Crit Care Res Pract.

[16] Schmittinger CA, Torgersen C, Luckner G, Schroder DC, Lorenz I, Dunser MW (2012). Adverse cardiac events during catecholamine vasopressor therapy: a prospective observational study. Intensive Care Med.

[17] Chawla LS, Ostermann M, Forni L, Tidmarsh GF (2019). Broad spectrum vasopressors: a new approach to the initial management of septic shock?. Critical Care.

